# Effects of taro [*Colocasia esculenta* (L.) Schott] slices on nutritional quality, sensory quality, and shelf life of Chinese pickled and steamed pork belly

**DOI:** 10.3389/fnut.2023.1290221

**Published:** 2023-11-10

**Authors:** Qinguo Quan, Yexuan Zhang, Asad Nawaz, Luya Feng, Zuodong Qin

**Affiliations:** ^1^Hunan Engineering Technology Research Center for Comprehensive Development and Utilization of Biomass Resources, College of Chemistry and Bioengineering, Hunan University of Science and Engineering, Yongzhou, China; ^2^School of Food Science and Engineering, Central South University of Forestry and Technology, Changsha, China

**Keywords:** fatty acid, lipid oxidation, protein oxidation, shelf life, *Colocasia esculenta* (L.) schott, pickled and steamed pork belly

## Abstract

This study aimed to investigate the influence of different ratios of taro slices (TS) on the nutritional quality, sensory quality, and shelf life of Chinese pickled and steamed pork belly (CPSPB). The study examined various aspects of CPSPB, including its proximate components, fat oxidation, fatty acid composition, protein hydrolysis, oxidation reaction, and induction period (IP). Additionally, the sensory quality and texture analysis were compared simultaneously. The results showed that the addition of TS to CPSPB significantly improved water and lipid loss (*p* < 0.05), increased the unsaturated/saturated ratio of fatty acids, and reduced lipid and protein oxidation. Additionally, the incorporation of TS extended the IP and enhanced the shelf life of CPSPB. Particularly, the addition of a specific amount of TS (60%) to CPSPB resulted in the highest organoleptic quality. Therefore, these results emphasize the positive impact of TS on the overall quality of CPSPB, highlighting its potential to enhance the nutritional value, sensory attributes, and shelf life.

## 1. Introduction

Chinese pickled and steamed pork belly (CPSPB), also known as kou rou in China, holds a significant place as a traditional dish among the Chinese population (1–3). The industry of prepared dishes in China is currently experiencing rapid development in both provincial and urban areas, with a focus on crucial aspects, such as quality, flavor, nutritional safety, traceability, and technical equipment (4). The CPSPB production processes use natural raw materials, mainly pork belly, similar to other traditional meat dishes. These are accompanied by vegetable ingredients like dried mustard, fermented soybean, and taro (3, 5, 6). Chinese pickling aims to enhance the flavor and prolong the shelf life of food by employing various methods (6). Steaming, as a hot processing technique, serves the dual purpose of sterilization and enhancing the formation of aromatic compounds (7). However, it is important to note that commercially available CPSPB currently exhibit variations in quality, have a limited shelf life, and lack standardized processing methods. Therefore, addressing these issues is of utmost importance to facilitate the efficient industrial production and meet the growing consumer demand for CPSPB.

Taro [*Colocasia esculenta* (L.) Schott] is primarily cultivated in the southern province of China (8–10). Moreover, it is grown in various regions and countries including the Caribbean, Hawaii, the Solomons, American Samoa, Western Samoa, the Philippines, Fiji, Sri Lanka, India, Nigeria, Indonesia, New Hebrides, Tonga, Niue, Papua New Guinea, and Egypt (11). The primary chemical components of it are starch, with smaller amounts of protein and lipids, along with trace mineral elements and vitamins (12). CPSPB with taro slices (TS) is a popular Chinese delicacy. It consists of pork belly, taro slices, and various additives, which are then steamed. The resulting steamed dish offers a delightful taste and a satisfying sensation of fullness. However, there is a lack of published research investigating the effects of TS on CPSPB. Specifically, the impact of TS on the chemical composition of CPSPB, which is closely associated with its nutritional, sensory, and preservative properties, remains unknown.

Frozen meat deteriorates during storage due to the degradation of lipids and proteins, as reported by Ali et al. (13). The oxidation and hydrolysis reactions of these components are critical in determining the quality and shelf life of meat and meat-based products. Oxidative reactions occur during various stages of meat processing, such as mincing, cooking, and salting, as well as during storage and processing of meat and meat-base products. These reactions promote the formation of reactive oxygen species (ROS) and increase the oxidative susceptibility of the end products (14). Therefore, it is important to scavenge ROS, in order to inhibit or suppress lipid and protein peroxidation and oxidation, TS contain antioxidant compounds, such as phenolic compounds (polyphenols chlorogenic acid, catechin, epicatechin, epigallocatechin (flavan-3-01s), gallic acid and proanthocyanidins), resistant starch, and polysaccharides (15, 16), which have the potential to scavenge ROS. Therefore, it is necessary to assess the oxidation of lipids and proteins, including the fatty acid composition of CPSPB to evaluate the effectiveness of TS in meat-based products.

To determine the optimal amount of TS used to interfere with CPSPB nutritional quality, sensory quality, and shelf life. The purpose of this study was to determine the proximate components, fat oxidation, fatty acid composition, protein hydrolysis, and oxidation reaction, as well as the induction period (IP) of CPSPB under the addition of 0%–100% mass ratio TS. The sensory quality and texture analysis of these samples were concurrently compared. In line with this, the findings of this study are intended to serve as a valuable reference for domestic and industrial production, providing insights into the benefits of incorporating TS into CPSPB. Additionally, these results can be utilized as a foundation for expanding the utilization of TS, with potential economic benefits in various applications.

## 2. Materials and methods

### 2.1. Raw materials and sample preparation

Taro and pork belly with a lean-to-fat ratio of approximately 1:2 were purchased from a local food market in Yongzhou, Hunan Province, China. They were immediately transported in an ice box to the laboratory within 30 min after purchase. To ensure freshness, the taro and pork belly were stored in a plastic container with crushed ice at the bottom, maintaining a transport temperature of 5°C–10°C. The samples were prepared using traditional Chinese household cooking methods.

Firstly, the fresh pork belly was pre-cooked in boiling water for 2 min using a pork-to-water ratio of 1:5 (w/v). After removing any remaining pig hairs from the skin and rinsing off surface impurities and blood with tap water, approximately 200 grams of pre-cooked pork belly were placed in a bowl and cut into pieces measuring 2.5 cm × 3.5 cm × 0.8 cm (length × width × height). These slices were then pickled for 30 min in a pickling jar containing a solution composed of 2% soy sauce, 2% sugar, 2% salt, and 0.1% monosodium glutamate. Next, the taro was carefully selected, peeled, and then cut into fillets of the same size as the pork belly using a slicing machine (QN-200, Henan Qineng Machinery Equipment Co., Ltd., China). It was important for the taro slices to be of uniform size and show no signs of rot on the surface. Different amounts of taro slices, ranging from 0 to 200 g in increments of 40 g, were combined with 200 g of pork in each sample. Finally, all ingredients were thoroughly mixed to ensure that the sample was well-blended for testing purposes.

The samples were prepared and placed in a pressure cooker (Joyoung, China) for 15 min, where they were subjected to high-pressure steaming on an induction cooker (2,200 W). After the steaming process, the samples were left to cool for 5 min before the pressure cooker was opened and the samples were separated. The cooled samples were then either used for textural profile analysis and sensory evaluation or minced using a mincer (Midea, China) to ensure homogeneity and obtain representative samples for further analysis. Finally, the minced samples were stored at −20°C until analysis.

For all the steaming experiments, seven groups were formed. These groups include: (I) The control group, which consisted of raw pickled pork belly. Following this, (II) CPSPB alone was examined. Subsequently, CPSPB was treated with different concentrations of TS. These concentrations included (III) 20% TS, (IV) 40% TS, (V) 60% TS, (VI) 80% TS, and (VII) 100% TS. For each determination, three replicates were conducted for all the treatments.

### 2.2. Determination of proximate components

The determination of moisture, crude protein, ash, crude fat, and sodium chloride content followed the guidelines outlined in the AOAC methods (2006) and were followed precisely during the analysis. The standard reference numbers and brief descriptions of the determination methods were as follows: moisture content was determined by oven drying the samples at 105°C by AOAC 950.46. Crude protein was determined using the Kjeldahl method, wherein the nitrogen-to-protein conversion factor of 6.25 was applied, as specified in AOAC 928.08. Ash content was determined by heating 3 g samples in an oven at 550°C until a constant weight was achieved, following the procedure outlined in AOAC 920.153. Crude lipids was determined through Soxhlet extraction, as described in AOAC 991.36. Moreover, NaCl contents was determined by Mohr method (AOAC 935.43, 33.7.10: 2000). The parameters were measured using the appropriate units specified in the standard, such as percentages or milligrams per gram.

### 2.3. Determination of fatty acids

Total lipids were extracted from minced pork belly using the method of Wu et al. (17) with minor modifications. The minced pork belly was homogenized with a 2:1 chloroform-methanol solution (Tianjin Damao Chemical Reagent Co., Ltd., Tianjin, China) at a final dilution of 10 times the sample volume. The extraction was carried out in a water bath at 60°C for 15 min, followed by filtration. For conversion to methyl esters, a sulfuric acid-methanol solution (12.5%, w/v) (Tianjin Damao Chemical Reagent Co., Ltd., China) was used. The samples obtained were analyzed using a GC7890A gas chromatograph (Agilent) equipped with an autosampler, split/splitless injector, a silica capillary column (DB-23; 30 m length × 0.32 mm id × 0.25 μm film thickness; Agilent), and a flame ionization detector. To calibrate the analysis, a 37-fatty acid methyl ester (FAME) mixed standard (Sigma-Aldrich Co., LLC) was run under the same conditions.

The following GC conditions were used for the experiment: nitrogen was employed as the carrier gas at a flow rate of 9 mL/min and a pressure of 6.6016 psi. The split ratio was set to 5:1. The injector temperature was set to 270°C with an injection volume of 1 μL, while the temperature of the flame ionization detector was maintained at 280°C. The temperature ramp-up procedure was as follows: a five-minute duration at 120°C, followed by an increase to 190°C at a rate of 5°C/min and hold for 12 min. Then, there was a further increase to 210°C at a rate of 2.5°C/min, and the temperature was held at 210°C for 10 min.

### 2.4. Determination of lipid oxidation

The lipid oxidation coefficient was estimated using peroxide value (POV) determinations and thiobarbituric acid (TBA). The TBA determination method was adapted from Sorensen et al. (18) with minor modifications. To begin, 10 g of the ground sample were homogenized with 50 mL of 7.5% trichloroacetic acid (TCA) solution containing 0.1% ethylenediaminetetraacetic acid (EDTA) using a BRS-500-B homogenizer (Anhui Bojin Instrument Factory, China) at 15,000 rpm for 30 s. Next, a 5 mL filtrate obtained through pipette filtration was transferred to a stoppered plastic tube. After that, 5 mL of 0.02 M TBA aqueous solution was added. The sample was adequately mixed and then incubated in a water bath at 100°C for 1 h. Subsequently, it was cooled down in cold water. Using a UV spectrophotometer (model 912A1113; Thermo Fisher Scientific, Germany), the absorbance of the sample was measured at a wavelength of 532 nm. The TBA content of the sample was determined by converting it using the 1,1,3,3-tetraethoxypropane (TEP) standard curve. The results were expressed as milligrams of malondialdehyde (MDA) per kilogram of pork belly sample. The pork belly sample’s POV levels were evaluated using the AOAC method 965.33 and expressed in milliequivalents (meq) of peroxide per kilogram.

### 2.5. Determination of protein hydrolysis and oxidation

#### 2.5.1. Determination of soluble protein extraction and concentration

Two grams of minced pork belly were transferred to a pre-weighed centrifuge tube. Then, 10 mL of 20 mM sodium phosphate buffer (pH 6.8) was added to the tube. The mixture was homogenized twice with a homogenizer at 15,000 rpm and 4°C for 30 s. Afterward, the mixture was allowed to stand for 1 h, and subsequently filtered through Whatman No. 1 filter paper. The filtrate was stored at −20°C until further analysis. Finally, the protein concentration in the filtrate was determined using the BCA kit (Sigma), with bovine serum albumin used as the standard for calibration.

#### 2.5.2. Determination of protein hydrolysis

The degree of hydrolysis of the protein sample was assessed using the TNBS assay, as described by Adlernissen (19) with slight modifications. Initially, 20 μL of the protein extraction sample was mixed with 980 μL of sodium phosphate buffer (pH 8.0) in a centrifuge tube. Subsequently, 1 mL of 1.0% TNBS solution was added to the tube, which was then vortexed. The resulting mixture was heated in a water bath at 50°C for 60 min. After 60 min of the water bath reaction, 2 mL of 0.1 N hydrochloric acid was added to the sample. The absorbance of the mixed sample was measured at 320 nm using a UV spectrophotometer (912A1113; Thermo Fisher Scientific, Germany). To construct a standard curve, glycine was used as the standard substance. The degree of hydrolysis of the protein sample was then calculated based on the standard curve. Finally, the results were expressed as the number of free amino groups per gram of soluble pork belly protein.

#### 2.5.3. Determination of protein oxidation

Protein oxidation was assessed by measuring protein carbonyl and sulfhydryl levels. Protein carbonyl levels were determined using the method described by Ali et al. (13) with slight modifications. Briefly, 0.7 mL of the protein extract sample was mixed with 0.3 mL of 10 mmol/L 2,4-dinitrophenylhydrazine (DNPH) and allowed to stand for 1 h at room temperature. To facilitate protein precipitation and purification, three successive additions of 1 mL of 40% trichloroacetic acid (TCA) and 1 mL of an ethanol/ethyl acetate mixture (1:1) were made. The resulting protein was then dissolved in 3 mL of 6 mol/L guanidine hydrochloride. The absorbance at 370 nm, corresponding to carbonyl levels, and the protein content at 280 nm were measured using an ultraviolet spectrophotometer (model 912A1113; Thermo Fisher Scientific, Germany). Results are expressed as the ratio of the absorbance at 370 nm to that at 280 nm. For the determination of sulfhydryl content, the method of Srinivasan et al. (20) was modified. Briefly, 1 mL of protein sample extract was dissolved in 2 mL of sodium phosphate buffer (pH 8.0) in a plastic tube. Subsequently, 0.5 mL of 10 mmol/L 5,5′-dithiobis (2-nitrobenzoic acid) (DTNB) reagent was added to the mixture. The mixture was then incubated in the shade for 1 h. After calibrating the UV spectrophotometer using the phosphate buffer solution, the absorbance of the sample was measured at 412 nm. The sulfhydryl content was expressed as millimoles of total free sulfhydryl groups per gram of soluble pork belly protein.

### 2.6. Texture profile analysis

Texture profile analysis (TPA) was performed using a texture analyzer (Universal TA; Shanghai Tengba Instrument Technology Co., Ltd., China) equipped with a 5 mm diameter cylindrical probe according to Bourne’s method (21). Samples of pickled pork belly, measuring approximately 1.0 cm in length, width, and height, were prepared for each treatment. The samples were compressed to 50% of their original height at a crosshead speed of 1.0 mm/s for two cycles. Parameters such as hardness, gumminess, and chewiness were calculated for each sample.

### 2.7. Sensory evaluation

The evaluation team consisted of 16 members, evenly distributed between the sexes (8 men and 8 women), aged 20–25 years. All team members had received professional training in food science and sensory evaluation. For the sensory evaluation, quantitative descriptive analysis following the method established by Morita et al. (22) was used. Each team member was allocated 10 g of pork belly samples, which were weighed and randomly numbered. To ensure no residual sensory characteristics from previous samples, the samples were placed on a glass plate. The team then engaged in a group discussion to establish standardized sensory descriptions for four attributes: color, aroma, taste, and texture. Subsequently, each team member rated the samples on a scale of 1–25 for each attribute (Table 1). The entire evaluation process took place in separate compartments within the same room, under white light at a temperature of 22°C–25°C. Higher scores on the scale indicated better organoleptic quality of the samples, approaching the standard descriptions.

### 2.8. OXITEST analysis

The oxidation tests were performed using the OXITEST reactor (Velp Scientifica, Usmate, Milan, Italy) as described by Verardo et al. (24). Each chamber of the reactor was filled with 6 g of minced pork belly samples. The test temperatures were set at 80°C, 90°C, and 100°C, while the initial oxygen pressure was maintained at 6 atm. The minced pork belly samples were placed in the OXITEST reactor to determine the induction period (IP). The IP values were determined in triplicate for each treatment.

### 2.9. Statistical analysis

Results from all experiments were presented as mean ± standard deviation. These experiments were performed in triplicate, with a sample size of three (*n* = 3). Treatment effects were analyzed using SPSS 19.0 (SPSS Inc., Chicago, IL) and one-way analysis of variance (ANOVA). The separation of means was determined through Duncan’s multiple-range test. Statistical significance was defined as *p* < 0.05, while *p* < 0.01 was considered statistically highly significant.

## 3. Results and discussion

### 3.1. Proximate composition

Following the steaming process, the proximate composition of CPSPB was affected by TS, as shown in Table 2. Significant increases in moisture and crude protein contents of pork belly were observed (*p* < 0.05) due to the pressure difference from the high-pressure steaming system, which caused lipids melting and liquefaction, and inorganic salt dissolution. Conversely, there was a significant decrease in the total lipids, ash contents and NaCl contents (*p* < 0.05) to 32.28 ± 0.60%, 2.94 ± 0.03% and 1.97 ± 0.02%, respectively. The alteration in fat content appeared to have the most significant influence on the composition of other components, which aligns with previous studies (23, 25). The initial moisture, crude protein, total fat, ash, and NaCl contents of raw pork belly were 40.05 ± 0.76%, 20.46 ± 0.89%, 35.49 ± 1.42%, 3.12 ± 0.04%, and 2.24 ± 0.06%, respectively.

**Table 1:** Reference of sensory evaluation on CPSPB.

Items	Describes of different scores				
	Dislike (5)	Slightly dislike (10)	Nor like nor dislike (15)	Slightly like (20)	Like very much (25)
Color	Brown is not the dominant color	Local small black or white patches cause uneven coloration	Mostly brown with a smattering of black or white spots	Uniform brown	Glossy, full, brown color with no black or white
Aroma	The smell is distinct, pungent, and even nauseating	An unusual or off-flavor characterized by its raw, burnt, or abnormal taste	The smell of steamed pork and taro dish is a little incongruous, or there is a small amount of off flavors	The normal steamed pork and taro dish has no off flavors or abnormal smell	Characterized by the rich aroma of steamed pork and taro, without any burnt paste taste, raw meat taste, or other unusual smells
Taste	Difficult to gulp	Greasy, raw, or salty taste	The flavor is acceptable, but not enjoyable	Taro and pork exhibit a delicious, pure flavor without any unpleasant notes	The taste of taro and pork is harmonious, mellow, and delicious, devoid of any sour or bitter flavors
Texture	The pattern is generally scattered	A small amount of deformation or ground meat	Slightly harder or softer, with some incomplete grain	Clear and solid in structure, both soft and firm	Fat without greasiness, thin without being wooden, soft and sticky skin and flesh, and clear lines

**Table 2:** Effects of TS on proximate composition of CPSPB (g/100 g fresh weight).

Treatments	Contents (%)				
	Moisture	Crude protein	Total lipids	Ash	NaCl content
Control (raw)	40.05 ± 0.76^b^	20.46 ± 0.89^d^	35.49 ± 1.42^a^	3.12 ± 0.04^a^	2.24 ± 0.06^a^
0% TS	42.74 ± 0.95^a^	22.15 ± 0.91^d^	32.28 ± 0.60^b^	2.94 ± 0.03^b^	1.97 ± 0.02^b^
20% TS	40.12 ± 1.16^b^	26.14 ± 0.67^c^	29.61 ± 0.47^c^	2.65 ± 0.13^c^	1.40 ± 0.12^c^
40% TS	38.31 ± 1.29^b,c^	29.30 ± 1.04^b^	28.25 ± 0.92^c,d^	2.32 ± 0.05^d^	1.15 ± 0.03^d^
60% TS	36.98 ± 0.73^c^	32.36 ± 0.72^a^	27.04 ± 0.34^d^	2.26 ± 0.08^d,e^	1.12 ± 0.08^d^
80% TS	36.54 ± 0.54^c^	32.49 ± 1.13^a^	26.57 ± 0.73^d^	2.09 ± 0.03^e^	0.98 ± 0.02^d,e^
100% TS	36.37 ± 0.39^c^	32.84 ± 0.53^a^	26.74 ± 0.56^d^	2.14 ± 0.04^e^	0.90 ± 0.06^e^

Different letters within a column are significantly different (*p* < 0.05).

Additionally, there was a gradual decrease in the moisture, total lipid, sodium chloride, and ash contents of pork belly, leading to a gradual increase in total protein content. The inclusion of 60% or more TS demonstrated no significant difference (*p* > 0.05) in moisture, total lipid, and protein content. This indicates that the addition of 60% or more TS stabilized the major components’ content in the pork belly samples, possibly due to the physical barrier and diffusion-driven physicochemical properties of TS. It is plausible that some of the diffused salt from CPSPB may have transferred to the TS during the steaming process, leading to a reduction in the saltiness and graininess of the pork belly, thereby harmonizing its texture. Throughout the steaming process, the moisture contents of pork belly decreased from 42.74 ± 0.95% to 36.98 ± 0.73%, whereas the total lipids contents decreased from 32.28 ± 0.60% to 27.04 ± 0.34%. This reflects the capacity of TS to bind water and lipids, resulting in reduced moisture and lipid content of pork belly, thereby contributing to the extended shelf life of CPSPB.

### 3.2. Lipid oxidation

**Figure 1:**
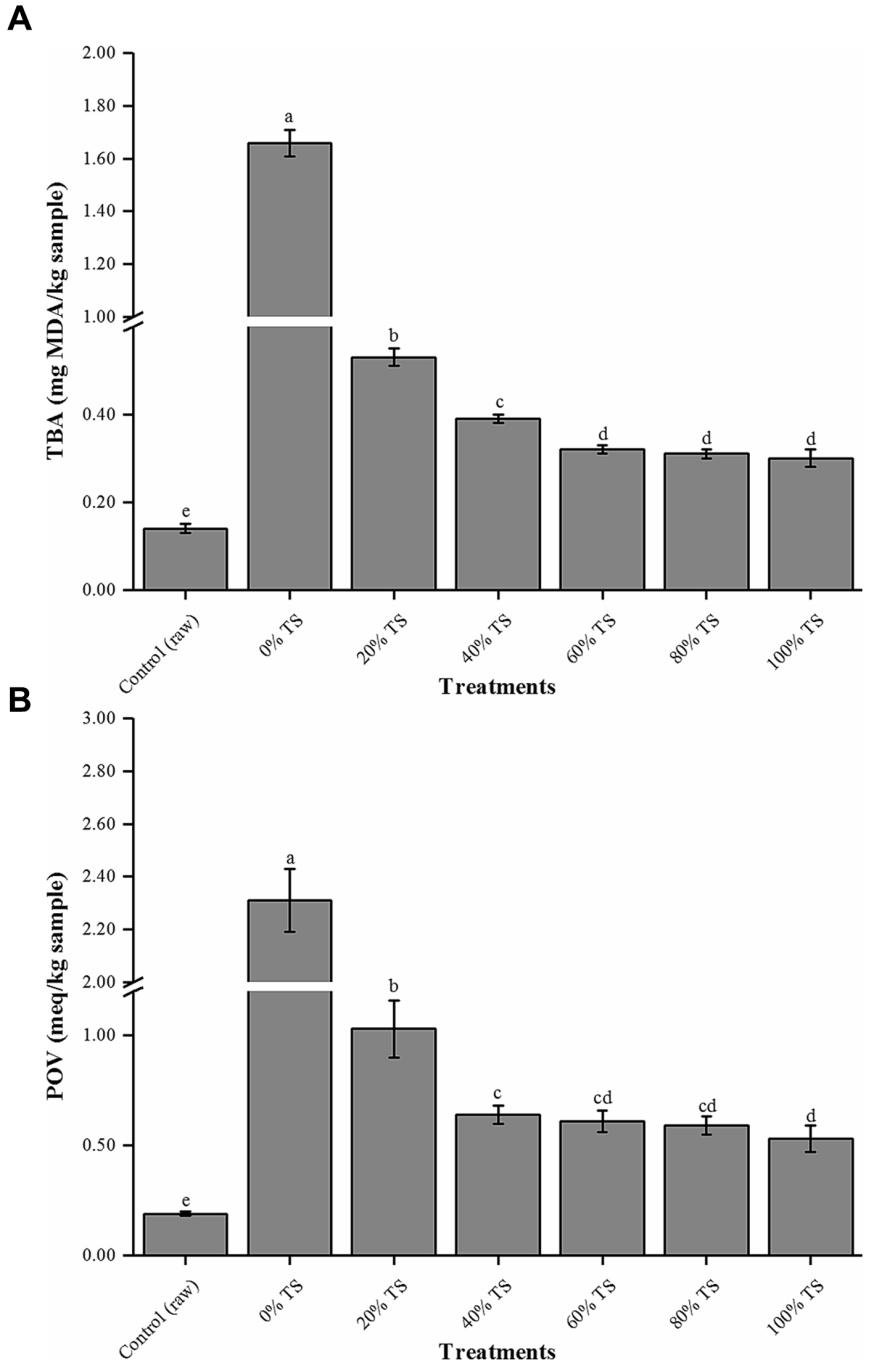
Levels of thiobarbituric acid (TBA, **A**) and peroxides levels (POVs, **B**) of CPSPB with different amounts of added of TS. Values are presented as mean ± SD. Bars with different letters are significantly different (*p* < 0.05).

Figure 1 illustrates that CPSPB samples treated with TS exhibited a significant decrease (*p* < 0.05) in both POV and TBA values compared to the original samples. This reduction can be attributed to the exposure of the pork belly samples to the antioxidant agents such as phenolic compounds, resistant starch, and polysaccharides (14, 15) present in TS, as well as the reduction in the exposed surface area of the pork belly to air, achieved by covering it with TS. The POV represents the number of primary oxidation products generated when ROS attack the double bonds of fatty acids. On the other hand, the TBA value measures the amount of secondary lipid oxidation products, which predominantly consist of aldehydes, known to contribute to off-flavors in meat products (21). Consequently, the addition of TS shows potential in extending the shelf life of CPSPB by effectively reducing the degree of lipid oxidation (26, 27).

### 3.3. Fatty acid profile

Table 3 demonstrates that the raw pork belly samples had the highest content of saturated fatty acids (SFA, 40.63 ± 0.16%), followed by monounsaturated fatty acids (MUFA, 37.01 ± 0.13%) and polyunsaturated fatty acids (PUFA, 22.36 ± 0.02%). The primary fatty acids in the raw samples were palmitic acid (C16:0, 23.06 ± 0.09%), stearic acid (C18:0, 16.13 ± 0.07%), oleic acid (C18:1n9c, 34.86 ± 0.14%), and linoleic acid (C18:2n6c, 18.27 ± 0.03%). These findings align with previous data obtained (3, 28, 29). Steaming resulted in a significant decrease in PUFA and a significant increase in MUFA and SFA (*p* < 0.05) in the pork belly samples. The increase in medium/short-chain MUFA and SFA may be attributed to the degradation of long-chain PUFA during autoclaving. However, Li et al. (30) demonstrated that prolonged cooking caused a significant decrease (*p* < 0.01) in the SFA content of pork, as it melted into the broth. Moreover, according to Zhang et al. (28), steaming did not significantly (*p* > 0.05) affect the fatty acid composition of grass carp fillets, but led to a slight increase in SFA. Thus, it is suggested that the specifics of the heating process, rather than the sample characteristics and heating method, may influence the fatty acid composition of meat products.

**Table 3:** Effects of TS on fatty acid profiles of CPSPB (area % of total fatty acids).

Retention time (min)	Fatty acid	Treatment						
		Control (raw)	0% TS	20% TS	40% TS	60% TS	80% TS	100% TS
8.57	C12:0	0.08 ± 0.00^a^	0.10 ± 0.01^a^	0.09 ± 0.01^a^	0.08 ± 0.02^a^	0.10 ± 0.01^a^	0.10 ± 0.01^a^	0.10 ± 0.01^a^
12.30	C14:0	0.86 ± 0.01^b^	0.89 ± 0.03^b^	0.87 ± 0.02^b^	0.91 ± 0.01^b^	0.98 ± 0.03^a^	1.01 ± 0.05^a^	0.99 ± 0.02^a^
15.45	C16:0	23.06 ± 0.09^c^	26.05 ± 0.47^a^	24.76 ± 0.47^b^	23.90 ± 0.55^b^	20.16 ± 0.18^d^	20.04 ± 0.06^d^	19.97 ± 0.11^d^
17.12	C17:0	0.32 ± 0.01^a^	0.30 ± 0.01^a^	0.29 ± 0.01^a^	0.25 ± 0.02^b^	0.20 ± 0.03^c^	0.13 ± 0.00^d^	0.13 ± 0.01^d^
18.23	C18:0	16.13 ± 0.07^a^	16.42 ± 0.63^a^	16.19 ± 0.20^a^	15.28 ± 0.46^b^	11.94 ± 0.31^c^	10.81 ± 0.03^d^	10.90 ± 0.21^c,d^
21.82	C20:0	0.24 ± 0.01^c^	0.26 ± 0.03^b,c^	0.27 ± 0.04^b,c^	0.30 ± 0.01^b^	0.36 ± 0.04^a^	0.33 ± 0.02^a,b^	0.30 ± 0.01^b^
	**∑SFA**	40.63 ± 0.16^b^	44.01 ± 1.05^a^	42.48 ± 0.49^a,b^	40.70 ± 0.34^b^	33.72 ± 0.15^c^	32.42 ± 0.02^c^	32.38 ± 0.07^c^
14.36	C15:1	0.11 ± 0.00^c^	0.20 ± 0.06^a^	0.19 ± 0.07^a^	0.15 ± 0.04^b^	0.10 ± 0.01^c^	0.08 ± 0.03^c,d^	0.06 ± 0.00^d^
16.20	C16:1	1.09 ± 0.04^c^	1.45 ± 0.03^b^	1.43 ± 0.01^b^	1.49 ± 0.03^a,b^	1.58 ± 0.02^a^	1.60 ± 0.01^a^	1.51 ± 0.00^a^
17.67	C17:1	0.18 ± 0.00^a^	0.17 ± 0.02^a^	0.15 ± 0.03^a,b^	0.14 ± 0.02^b^	0.11 ± 0.00^c^	0.10 ± 0.01^c^	0.09 ± 0.00^c^
19.31	C18:1n9c	34.86 ± 0.14^c^	38.02 ± 0.54^b^	38.87 ± 0.21^a,b^	39.74 ± 0.43^a,b^	40.37 ± 0.08^a^	40.30 ± 0.15^a^	40.39 ± 0.02^a^
23.09	C20:1	0.78 ± 0.01^b^	0.85 ± 0.02^a^	0.76 ± 0.05^b^	0.67 ± 0.03^c^	0.60 ± 0.01^c,d^	0.57 ± 0.04^d^	0.58 ± 0.03^d^
	**∑MUFA**	37.01 ± 0.13^c^	40.67 ± 0.25^b^	41.40 ± 0.15^a,b^	42.18 ± 0.38^a^	42.76 ± 0.34^a^	42.65 ± 0.11^a^	42.62 ± 0.01^a^
20.17	C18:2n6t	1.89 ± 0.01^b^	2.26 ± 0.06^a^	2.07 ± 0.13^a^	1.26 ± 0.13^c^	0.10 ± 0.01^d^	0.10 ± 0.00^d^	0.09 ± 0.01^d^
20.71	C18:2n6c	18.27 ± 0.03^b^	12.90 ± 0.13^e^	14.73 ± 0.21^d^	16.08 ± 0.30^c^	20.23 ± 0.12^a^	20.14 ± 0.05^a^	20.76 ± 0.02^a^
21.69	C18:3n3	0.82 ± 0.01^c^	0.73 ± 0.07^c^	0.81 ± 0.01^c^	0.95 ± 0.03^b^	1.34 ± 0.06^a^	1.37 ± 0.01^a^	1.39 ± 0.02^a^
24.32	C20:2	0.84 ± 0.01^b^	0.69 ± 0.01^c^	0.79 ± 0.03^b^	0.86 ± 0.08^ab^	0.96 ± 0.01^a^	0.94 ± 0.01^a^	0.93 ± 0.01^a^
25.73	C20:3n3	0.41 ± 0.01^c^	0.67 ± 0.04^a^	0.58 ± 0.13^b^	0.45 ± 0.02^c^	0.32 ± 0.01^d^	0.29 ± 0.01^d^	0.28 ± 0.02^d^
26.54	C20:4n6	0.13 ± 0.02^b^	0.07 ± 0.03^c^	0.14 ± 0.02^a,b^	0.18 ± 0.02^a^	0.20 ± 0.01^a^	0.18 ± 0.02^a^	0.20 ± 0.00^a^
	**∑PUFA**	22.36 ± 0.02^a^	17.32 ± 0.17^c^	19.12 ± 0.19^b^	19.77 ± 0.34^c^	23.15 ± 0.10^a^	23.02 ± 0.14^a^	23.65 ± 0.02^a^
	**∑UFA**	59.37 ± 0.12^b,c^	57.99 ± 0.28^c^	60.52 ± 0.18^b^	61.95 ± 0.43^b^	65.91 ± 0.28^a^	65.67 ± 0.10^a^	66.27 ± 0.00^a^
	**UFA/SFA**	1.46 ± 0.01^c,d^	1.32 ± 0.07^d^	1.42 ± 0.02^d^	1.52 ± 0.04^c^	1.95 ± 0.02^b^	2.03 ± 0.01^a^	2.05 ± 0.00^a^

The addition of various percentages of TS influenced the fatty acid composition of CPSPB in this study. The proportion of MUFAs (40.67 ± 0.25%-42.76 ± 0.34%) and PUFAs (17.32 ± 0.17%-23.65 ± 0.02%) gradually increased, while the proportion of SFAs (44.01 ± 1.05%-32.38 ± 0.07%) continued to decrease. The UFA/SFA ratio changed from 1.32 ± 0.07 to 2.05 ± 0.00 with the sequential addition of TS from 0 to 100% of the sample proportion. When TS exceeded 60% of the sample proportion, MUFAs constituted the majority of fatty acids compared to SFAs, while PUFAs remained the least abundant. The predominant fatty acid changes driving this trend were the decrease in C16:0, C17:0, and C18:0, and the increase in C18:1n9c, C18:2n6c, C18:3n3, and C20:2. Along with the Maillard reaction products (especially melanin) of CPSPB with TS, TS contains several naturally occurring antioxidant constituents that are absorbed by the samples during steaming (23, 31). They contribute to inhibiting the autoxidation of unsaturated fatty acids. As a result, delaying the steaming process leads to a significant decrease in PUFAs and a significant increase in MUFAs and SFAs. Furthermore, the presence of C18:1n9c, C18:2n6c, and C18:3n3 in taro can combine with CPSPB during the steaming process, thereby increasing the overall fatty acid content (11). It is interesting to observe that the changes in MUFA, PUFA, and SFA were not significant (*p* > 0.05) when 60% or more TS was added. The alterations in fatty acid composition were generally consistent with the lipid oxidation indices (POV and TBA) observed in this study, further supporting the potential of TS as an effective lipid antioxidant when added to CPSPB.

### 3.4. Protein hydrolysis and oxidation

**Figure 2:**
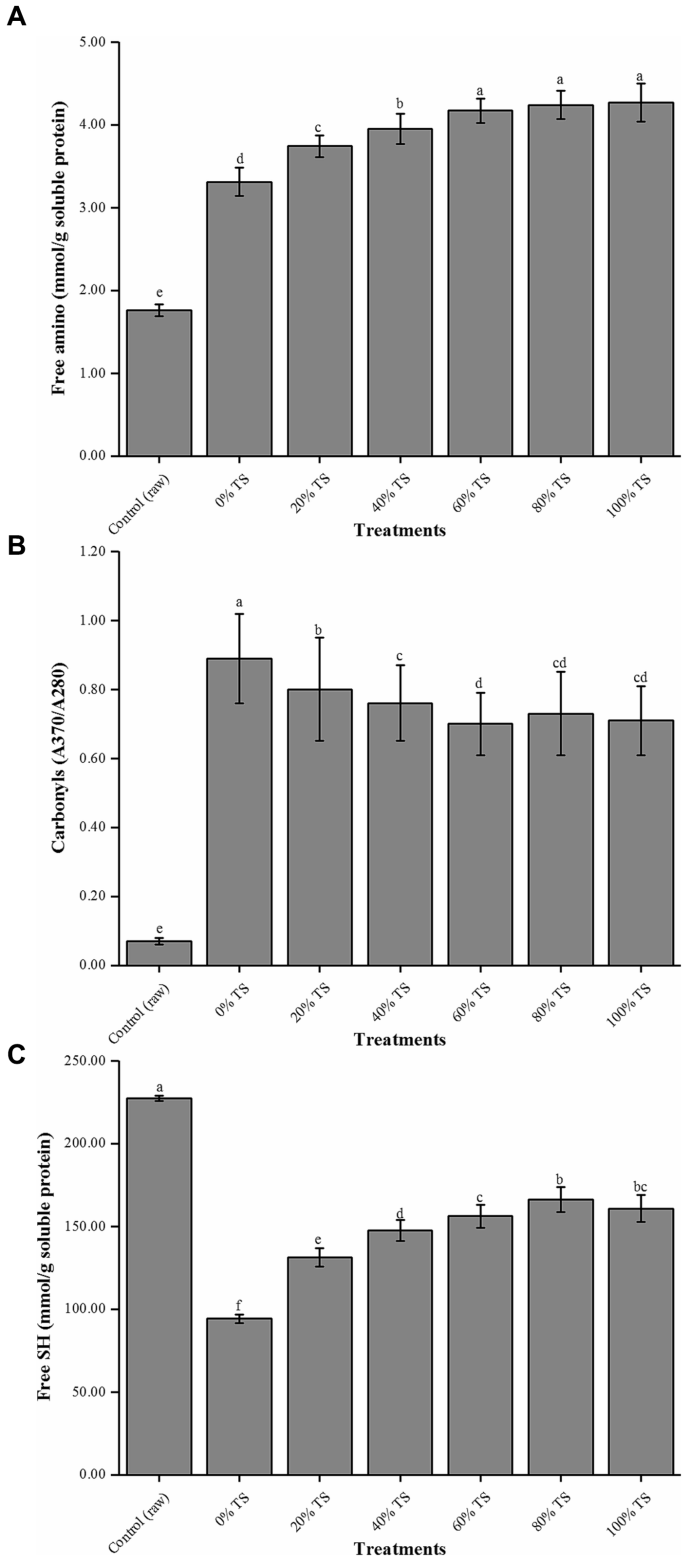
Free amino **(A)**, carbonyl **(B)** and free SH values **(C)** of CPSPB with different levels of TS added. Values are expressed as mean ± SD. Bars with different letters are significantly different from each other (*p* < 0.05).

The results demonstrated a significantly lower content of free amino groups in raw pork belly compared to the other treatments (*p* < 0.05) (Figure 2A). This suggests that the application of high temperature during cooking led to thermal denaturation of the proteins, which may explain the observed increase in free amino acid content in the tested samples (32). Additionally, the content of free amino acids displayed a concentration-dependent response to the addition of TS. Increasing the TS concentration from 0 to 60% resulted in a significant increase in free amino acid content from 3.31 ± 0.17 to 4.17 ± 0.15 mmol/g soluble protein (*p* < 0.05). However, further increasing the TS additions to 80% and 100% did not significantly influence the free amino acid content (*p* > 0.05). These findings suggest that within a certain range, TS positively affects the hydrolysis process of pork belly proteins. This beneficial effect can be attributed to the Maillard reaction that takes place between the glucose present in TS and the hydrolysate of pork belly protein during the steaming process, facilitating the hydrolysis reaction.

To evaluate the degree of protein oxidative degradation in the samples, carbonyl (Figure 2B) and free sulfhydryl (SH; Figure 2C) counts were measured (13). Steaming significantly promoted the oxidation of proteins in the samples (*p* < 0.05). The carbonyl value increased from 0.07 ± 0.01 to 0.89 ± 0.13, while free SH decreased from 227.41 ± 1.59 to 94.25 ± 2.61 mmol/g soluble protein. These findings align with previous studies that reported higher protein oxidation in cooked samples compared to raw samples exposed to different time/temperature cooking combinations (33, 34). Although a slight negative correlation was observed between the amount of TS added and the degree of protein oxidation, both values were significantly higher than those of the raw samples (*p* < 0.05). Among all TS treatments, the lowest carbonyl value was 0.71 ± 0.10 (100% TS), and the highest free SH was 166.13 ± 7.46 mmol/g soluble protein (80% TS). These results, consistent with the findings on fat oxidation in this study, strongly suggest that the addition of TS affects protein oxidation in pork belly. It is worth noting that adding more than 60% TS did not contribute to further improvements in the protein oxidation of CPSPB. Therefore, it is plausible that protein oxidation occurs concurrently with lipid oxidation, as previous reports indicated the relationship between the primary and secondary lipid oxidation products as substrates for protein oxidation (35, 36). Moreover, the inhibitory effect of TS on lipid oxidation may explain the lower level of protein oxidation observed in CPSPB (13).

### 3.5. Sensory quality

**Figure 3:**
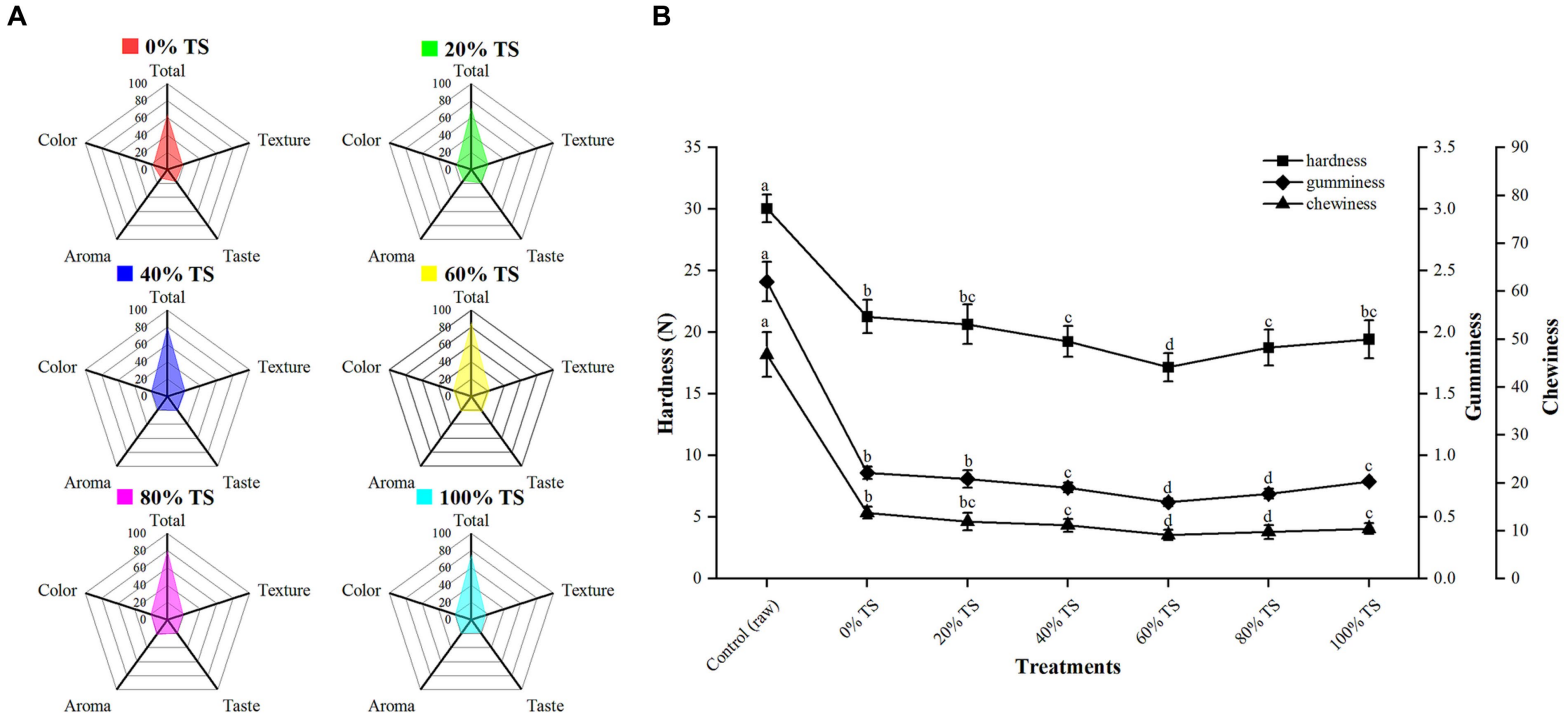
Sensory parameters **(A)** and TPA **(B)** of CPSPB with different levels of added TS. Values are presented as mean scores of each attribute.

The addition of varying levels of TS significantly improved the sensory quality (color, aroma, flavor, and texture) of CPSPB compared to the untreated samples (*p* < 0.01) (Figure 3A), as demonstrated by the sensory evaluation conducted by the panelists. The untreated pork belly received the lowest mean score (TS = 64.00), indicating its inferior sensory attributes. The incorporation of TS with CPSPB resulted in a pronounced flavor profile and contributed to the desirable color, aroma, and flavor of the pork belly samples. The total mean score increased from 71.30 to 85.78 as the percentage of TS increased from 20% to 60%, but decreased to 74.79 when 100% TS was added. This trend was also observed for the four sensory quality attributes. The samples with the highest mean scores for each attribute (color = 22.03, aroma = 20.65, taste = 21.26, texture = 21.84) and a total mean score of 85.78 displayed the most favorable sensory characteristics when 60% TS was added to CPSPB. Conversely, when TS was added at lower levels (<60%), there was no significant reduction in the brown color of CPSPB, nor was the full combination of TS and belly able to produce a noticeable strong flavor perceived by the panelists. Furthermore, the presence of relatively high lipid content in the CPSPB, which was insufficient to be absorbed by TS, resulted in a pronounced greasiness that could be overwhelming to the senses. Similarly, the aroma and flavor of CPSPB were not distinctly perceived, and the color became lighter and somewhat earthy when excessive TS (>60%) was added. The excessive loss of moisture and fat led to a tough texture. These findings align with the results obtained by Lorido et al. (37), who reported that the texture of a product is often influenced by its composition, specifically moisture and intramuscular lipid content. Moreover, by assessing the hardness, gumminess, and chewiness of the CPSPB at different TS levels, it was observed that the lowest values were achieved with the addition of 60% TS, resulting in soft, smooth, and tender CPSPB (Figure 3B). In conclusion, the addition of an appropriate level of TS (60%) yielded the most favorable organoleptic qualities in CPSPB (see Figure 3).

### 3.6. Shelf life

The OXITEST reactor is characterized by its simplicity, as it does not require sample pretreatment (24). It achieves predicting the shelf life of lipid-rich foods by measuring the oxygen consumption required to oxidize fats and oils. The reactor offers a rapid and accurate process for determining the rancidity of fats and oils. Moreover, this method allows for the complete assessment of fat and oil rancidity without the need to isolate lipids from the food matrix. Additionally, the OXITEST reactor enables the prediction of the induction period (IP) of the autoxidation process for different foods at various temperatures.

The IP values for CPSPB from different treatments at three induction temperatures: 80°C, 90°C, and 100°C are presented in Table 4. The observed behavior showed that the natural logarithm of IP displayed a linear relationship with increasing induction temperature (*R*^2^ > 0.99). These lines exhibited negative slopes and positive intersections, indicating that as temperature rose, the IP decreased, resulting in reduced oxidative stability and shorter preservation times for the samples. These findings support previous reports (35, 38) that storing meat at low temperatures can help prevent fat and protein oxidation during frozen storage.

**Table 4:** Effects of TS on induction period (IP) of CPSPB.

Treatments	IP (h)—T (°C)	*R* ^2^	Estimated IP25 (days:hour:min)
Control	ln(IP) = −0.090213 T + 8.971427	0.9987	34:09:36
0% TS	ln(IP) = −0.086359 T + 8.405716	0.9991	21:12:20
20% TS	ln(IP) = −0.088567 T + 9.692105	0.9972	73:16:35
40% TS	ln(IP) = −0.090273 T + 10.022361	0.9959	98:05:55
60% TS	ln(IP) = −0.092546 T + 10.167948	0.9992	107:08:47
80% TS	ln(IP) = −0.093695 T + 10.328024	0.9989	122:10:29
100% TS	ln(IP) = −0.094723 T + 10.491137	0.9993	140:11:20

To estimate the IP at room temperature (25°C), a linear model derived from the OXITEST reactor calculations was employed. The IP at 25°C (IP 25) for CPSPB without added TS was found to be shorter (IP 25 = 21:12:20, days: hours: minutes) compared to the raw product (IP 25 = 34:09:36, days: hours: minutes). This discrepancy may be due to the induction of fat and protein oxidation during the heating process, as cooked samples generally exhibit lower oxidative stability than raw meat (39). With increasing TS addition (from 20% to 100%), the IP 25 values increased (from 73:16:35 to 144:11:20, days: hours: minutes), indicating an extended storage period for the samples. Furthermore, the addition of TS affected the IP values, as evidenced by changes in the slope of the linear model. These results align with the findings of fat and protein oxidation in this study, highlighting the positive impact of TS treatment on the oxidative stability of CPSPB.

## 4. Conclusion

Trao (TS) plays a pivotal role in enhancing the nutritional quality, sensory attributes, and shelf life of CPSPB, as a precursor to its industrial production. Our study revealed that the addition of TS resulted in the reduction of moisture content, total fat content, ash content, and NaCl content, while simultaneously increasing the crude protein content of CPSPB. Furthermore, our findings confirmed that TS effectively mitigated fat oxidation in CPSPB, as evidenced by the decreased levels of TBA and POV, along with an elevated ratio of UFA to SFA. Additionally, TS exhibited a certain degree of promotion of protein hydrolysis and inhibition of protein oxidation in CPSPB. The OXITEST analysis demonstrated a prolonged shelf life of CPSPB when higher levels of TS were added at the same induction temperature. Moreover, the chemical composition changes resulting from the addition of TS significantly influenced the organoleptic and nutritional qualities of CPSPB. While the inclusion of 100% TS maximized the shelf life of the samples, the addition of 60% TS yielded the best organoleptic quality of CPSPB, as determined by TPA and sensory evaluations. These results provide a solid foundation for investigating the preservation mechanisms facilitated by TS. More research is required to study changes in odor and taste and determine the optimal organoleptic properties of CPSPB prepared using a 60% TS.

## Data availability statement

The original contributions presented in the study are included in the article/supplementary material, further inquiries can be directed to the corresponding author.

## Author contributions

QQ: Data curation, Methodology, Software, Writing – original draft, Writing – review & editing. YZ: Data curation, Software, Writing – original draft. AN: Methodology, Writing – review & editing. LF: Writing – review & editing. ZQ: Funding acquisition, Project administration, Resources, Supervision, Validation, Writing – review & editing.
